# The concomitants of conspiracy concerns

**DOI:** 10.1007/s00127-017-1354-4

**Published:** 2017-03-29

**Authors:** Daniel Freeman, Richard P. Bentall

**Affiliations:** 1 Department of Psychiatry, University of Oxford, Warneford Hospital, Oxford, UK; 2 Department of Psychological Sciences, Institute of Psychology, Health, and Society, University of Liverpool, Waterhouse Block B, Liverpool, UK

**Keywords:** Conspiracy, Paranoia, Mistrust, Epidemiology

## Abstract

**Purpose:**

A conspiracy world view may be a form of mistrust that is typically corrosive to individual and societal well-being. Our aim was to establish the correlates of conspiracy thinking in an epidemiologically representative sample.

**Methods:**

US National Comorbidity Survey-Replication (NCS-R) data were analysed from 5645 people who had completed the item “I am convinced there is a conspiracy behind many things in the world.” Results were weighted to be representative of the US adult English speaking household population.

**Results:**

1618 people (weighted 26.7%) endorsed the conspiracy belief item. These individuals were more likely to be: male; currently unmarried; less educated; in a lower income household; outside the labour force; from an ethnic minority group; not attending religious services; taking a weapon outside; and perceiving themselves as of lower social standing compared to others. Individuals endorsing the conspiracy belief item had lower levels of physical and psychological well-being, higher levels of suicidal ideation, weaker social networks, less secure attachment style, difficult childhood family experiences, and were more likely to meet criteria for a psychiatric disorder. There were no differences between those who endorsed conspiracy beliefs and those who did not in age, importance of religious beliefs in daily life, body mass index, or in having a gun at home.

**Conclusions:**

Viewing conspiracies in the world is associated with a raised risk of a wide range of adverse circumstances. It is a type of cognitive style that requires systematic empirical study, including monitoring of prevalence, tests of causation, and modelling of propagation.

## Introduction

It is difficult to overestimate the role of belief systems in human affairs. For example, political ideologies, which serve a variety of psychological functions [1], have provoked the most profound historical events, as have religious belief systems, which continue to impact on political life globally [2]. Conspiracy theories are an important type of belief system, which have often had negative historical effects, for example when they have fuelled violent ideologies (as when the stab-in-the-back myth was used to attribute German defeat in the First World War to a conspiracy of Jews and communists) or have been damaging to human well-being in other ways (for example, when the belief that the AIDS virus had been manufactured in American laboratories impeded the implementation of effective treatments in South Africa). However, these types of beliefs have been subjected to only limited empirical study.

Our interest is in ‘false conspiracy theories’ [3], of which there are many. These include, for example, world conspiracies (e.g., concerning Jews, a new world order, aliens), event conspiracies (e.g., concerning UFOs, moon landings, 9/11), technology conspiracies (e.g., about surveillance, the suppression of technologies) and disease conspiracies (e.g., creation of AIDS, chemtrail theory, the alleged link between vaccination and autism). We consider these theories to have four common characteristics: the world or an event is held to be not as it seems; there is believed to be a cover-up by powerful others; the believer’s explanation of events is accepted only by a minority; and the explanation is unsupported when the evidence is weighed up. Our interest is in clearly unfounded ideas.

We consider that conspiracy beliefs have close ties with the paranoia spectrum—in which a person perceives direct threats to themselves from others—that we have studied extensively [4, 5]. Unfounded conspiracy beliefs and paranoid ideas are both forms of excessive mistrust that may be corrosive at both an individual and societal level. In previous work analysing epidemiological surveys, we have found that paranoia is associated with youth, lower intellectual functioning, being single, poverty, poor physical health, poor social functioning, less perceived social support, disrupted attachment experiences in childhood, stress at work, less social cohesion, less calmness, less happiness, suicidal ideation, and a great range of other psychiatric symptoms [6, 7]. Empirical research on conspiracy beliefs is in its infancy and we are unaware of a similarly comprehensive investigation of their correlates.

There is, however, growing awareness of the importance of conspiracy beliefs and research has started into their psychological basis. Oliver and Wood [8], using data from four US nationally representative election surveys, report that half of the US population endorses at least one conspiracy belief, though approximately half of those individuals endorse one such belief only (i.e., a quarter of the total). They found conspiracy beliefs were more likely to be held by less educated respondents and African Americans. Lewandowsky et al. [9] carried out an online survey of over 1000 people and concluded that ‘conspiratorial thinking contributes to the rejection of science’ such as the overwhelming research consensus that human activity is affecting the climate. A similar conclusion that conspiracist ideation erodes trust in science was reached in an internet panel survey of a 1000 people in the US [10]. An experimental study with students indicated that exposure to conspiracy beliefs may reduce engagement in politics [11], while the presence of paranoia and the holding of conspiracy theories were significantly associated in a study of 120 students [12]. In a study of almost 2000 people in Britain, there was an association of conspiracy thinking with lower self-esteem and more negative attitudes to authority [13]. Brotherton and French [14] found that people who have a conspiracist view are particularly susceptible to the ‘conjunction fallacy’, overestimating the likelihood of co-occurring events.

Given the potential consequences of conspiracy thinking, we carried out a secondary analysis of relevant data collected during a US mental health survey that conducted face-to-face interviews with a large representative adult population. The prediction, based upon our paranoia research, was that a conspiracist worldview would be associated with numerous indicators of poorer social, psychological, and physical health. That is, conspiracy interpretations of the world would flourish in the context of marginalisation, poverty, adverse childhood experiences, lack of control, low self-esteem, and unhappiness. We believe that this is the most comprehensive report to date of the concomitants of conspiracy thinking.

## Methods

### Participants

The National Comorbidity Survey-Replication (NCS-R) was a US nationally representative face-to-face household cross-sectional psychiatric survey conducted between February 2001 and April 2003. Full details can be found in Kessler et al. [15, 16]. English speaking adults (age 18 and older) were selected from a multistage clustered area probability sample of non-institutionalised civilian households. The current report focuses upon those who completed both Parts I and II of the NCS-R, which was 5692 of the 9282 total NCS-R respondents.

### Assessments

All items from the NCS-R survey instrument can be seen at: http://www.hcp.med.harvard.edu/ncs/replication.php. As described by Kessler et al. [15], the NCS-R survey instrument principally comprised the World Health Organization (WHO) Composite International Diagnostic Interview (CIDI) developed for the WHO World Mental Health (WMH) Survey Initiative. Additional sections were also included for the US survey. The key item assessing the presence of conspiracy beliefs was from Section 16 (Personality) (International Personality Disorders Examination) [17], included in part II of the NCS-R instrument: “I am convinced there is a conspiracy behind many things in the world” (PEA83).

### Analysis

All analyses were carried out using the complex survey commands of SPSS version 22 with the part II weights of the NCS-R applied. General linear models or multinomial logistic regressions were carried out to test correlates of conspiracy concerns. Covariates were deliberately not used. The aim was to establish the strength of association of single variables with conspiracy beliefs, not to try to determine the unique contribution of each variable. There are numerous cautions in the literature against inappropriate use or interpretations of covariates especially in non-randomised studies [18]. The only exception was that a number of analyses were repeated controlling for paranoia (“Did you ever believe that there was an unjust plot going on to harm you or to have people follow you that your family and friends did not believe was true?”), an item completed by a smaller number of survey respondents, in order to demonstrate the effects were not simply due to this stronger form of personalised mistrust. All hypothesis testing was two-tailed.

## Results

### Prevalence of belief in conspiracy

1618 people (weighted 26.7%) endorsed the conspiracy belief item, and 4027 people (weighted 73.3%) did not. Table 1 shows the associations with socio-demographic factors. Men were more likely to endorse the conspiracy item than women. There was no statistically significant difference in age between those who endorsed the conspiracy item (estimated mean age = 45.8, std. error = 0.729) and those who did not (estimated mean age = 44.7, std error = 0.497), *t* = −1.457, *df*  = 42.000, *p* = 0.153. Not being currently married, lower levels of education, being outside of the labour force, being in particular ethnic minority groups (e.g. African American, Hispanic), and low religious attendance were all associated with a belief in conspiracy. Household income was lower in those who endorsed conspiracy beliefs (estimated mean income = $47,193, std. error = 1580.7) than those who did not (estimated mean income = $63,824, std. error = 1728.0), *t* = 29.86, *df* = 42.00, *p* < 0.001. People who endorsed the conspiracy belief item were also more likely to report that in the past year they were hungry but could not afford food.

**Table 1:** Socio-demographic factors

Variable	Conspiracy belief (*n*) (weighted percentage)	Not endorsing conspiracy belief (*n*) (weighted percentage)	Odds ratio	95% CI	*p* value
Sex					
Female	862 (24.5%)	2417 (75.5%)			
Male	756 (29.2%)	1610 (70.8%)	1.27	1.06, 1.54	0.013
Marital status					
Married	839 (24.1%)	2375 (75.9%)			
Never married	394 (29.0%)	816 (71.0%)	1.28	1.07, 1.54	0.009
Divorced/separated/widowed	385 (30.9%)	836 (69.1%)	1.41	1.14, 1.75	0.003
Years in education					
Greater than or equal to 16	224 (13.7%)	1191 (86.3%)			
13–15 years	435 (22.6%)	1265 (77.4%)	1.83	1.31, 2.55	0.001
12 years	584 (31.6%)	1112 (68.4%)	2.90	2.16, 3.88	<0.001
0–11 years	375 (42.2%)	459 (57.8%)	4.59	3.37, 6.26	<0.001
Work status					
Employed	977 (23.4%)	2768 (76.6%)			
Not employed	77 (29.9%)	202 (70.1%)	1.40	0.92, 2.12	0.114
Not in labour force	556 (32.8%)	1053 (67.2%)	1.60	1.36, 1.89	<0.001
Race					
Non-Latino white	997 (22.2%)	3149 (77.8%)			
All other Asian	27 (28.3%)	55 (71.7%)	1.38	0.78, 2.47	0.265
Mexican	128 (37.8%)	216 (62.2%)	2.14	1.40, 3.26	0.001
All other Hispanic	67 (37.3%)	112 (62.7%)	2.09	1.29, 3.39	0.004
Afro-Caribbean	15 (35.1%)	22 (64.9%)	1.90	0.77, 4.69	0.158
African American	316 (41.5%)	357 (58.5%)	2.49	1.89, 3.29	<0.001
All other	68 (38.2%)	116 (61.8%)	2.17	1.31, 3.61	0.004
Religious attendance					
Never	381 (33.6%)	695 (66.4%)			
Less than once a month	407 (25.7%)	1054 (74.3%)	0.69	0.55, 0.85	0.001
1-3 times a month	213 (24.3%)	540 (75.7%)	0.63	0.45. 0.90	0.013
Once a week	293 (23.1%)	904 (76.9%)	0.59	0.44, 0.79	0.001
More than once a week	168 (27.0%)	458 (73.0%)	0.73	0.53, 1.01	0.054
Importance of religious beliefs:					
Not at all important	127 (26.4%)	314 (73.6%)			
Not very important	139 (23.3%)	434 (76.7%)	0.85	0.56, 1.29	0.435
Somewhat important	454 (26.5%)	1145 (73.5%)	1.01	0.72, 1.40	0.966
Very important	896 (27.4%)	2124 (72.6%)	1.05	0.77, 1.45	0.745
In the past 12 months, were you ever hungry, but didn’t eat because you could not afford enough food?					
No	1291 (27.3%)	3079 (72.7%)			
Yes	115 (43.8%)	134 (56.2%)	2.08	1.47, 2.93	<0.001
Taken gun outside (past 30 days)					
No	1531 (26.3%)	3872 (73.7%)			
Yes	86 (33.8%)	150 (66.2%)	1.43	0.99, 2.06	0.059
Taken other weapon outside (past 30 days)					
No	1413 (25.5%)	3731 (74.5%)			
Yes	202 (38.7%)	290 (61.3%)	1.84	1.42, 2.40	<0.001
Gun at home					
No	1094 (27.6%)	2610 (72.4%)			
Yes	485 (25.0%)	1327 (75.0%)	0.87	0.74, 1.03	0.110

Respondents were also asked to rate themselves on ladders relative to other people in the United States and their community: “At the top of the ladder are the people who are the best off—those who have the most money, the most education and the most respected jobs. At the bottom are the people who are the worst off—who have the least money, least education, and the least respected jobs or no job. The higher up you are on the ladder, the closer you are to the people at the very top; the lower you are, the closer you are to the people at the very bottom. Please place a large “X” on the rung where you think you stand at this time in your life, relative to other people in the US”; “People define community in different ways; please define it in whatever way is most meaningful for you. At the top of the ladder are the people who have the highest standing in their community. At the bottom are the people who have the lowest standing in their community. Please place a large “X” on the rung where you think you stand at this time in your life, relative to other people in your community”. Those with a belief in conspiracy rated themselves lower on the US ladder (estimated mean = 5.66, std. error = 0.70) than those who did not endorse the conspiracy item (estimated mean = 6.23, std. error = 0.048), *t* = 7.91, *df* =  42.00, *p* < .001. Individuals with a belief in conspiracy (estimated mean = 6.14, std. error = 0.078) also rated themselves lower in their communities than individuals who did not endorse the conspiracy item (estimated mean = 6.64, std error = 0.048), *t* = 6.58, *df* = 42.00, *p* < .001.

### Physical and psychological Well-being

Tables 2 and 3 display data on the physical and psychological health of the population. In general physical health is poorer in people who hold conspiracy beliefs, while there is clearly lower psychological well-being over the past 30 days. The endorsement of the conspiracy item was highly associated with the specific paranoia psychosis item (Did you ever believe that there was an unjust plot going on to harm you or to have people follow you that your family and friends did not believe was true?), odds ratio = 7.81, 95% CI = 3.40, 17.93, *p* < .001. We therefore repeated the analyses in Table 3 controlling for paranoia, but all significant associations remained.

**Table 2:** Physical health

Variable	Conspiracy belief (*n*) (weighted percentage)	Not endorsing conspiracy belief (*n*) (weighted percentage)	Odds ratio	95% CI	*p* value
BMI					
Healthy weight (18.5–24.9)	563 (26.2%)	1510 (73.8%)			
Underweight (<18.5)	60 (29.5%)	125 (70.5%)	1.18	0.69, 2.02	0.546
Overweight (25.0–29.9)	498 (26.3%)	1308 (73.7%)	1.01	0.81, 1.25	0.963
Obesity class I (30.0–34.9)	289 (28.5%)	616 (71.5%)	1.12	0.85, 1.48	0.406
Obesity class II (35.0–39.9)	102 (24.1%)	248 (75.9%)	0.89	0.63, 1.27	0.520
Obesity class III (>40)	77 (29.2%)	152 (70.8%)	1.16	0.80, 1.67	0.427
Arthritis/rheumatism					
No	1114 (25.1%)	2952 (74.9%)			
Yes	502 (30.9%)	1070 (69.1%)	1.33	1.11, 1.60	0.003
Chronic back/neck problems					
No	979 (24.3%)	2777 (75.7%)			
Yes	639 (32.4%)	1250 (67.6%)	1.49	1.27, 1.74	<0.001
Stroke					
No	1563 (26.5%)	3932 (73.5%)			
Yes	54 (31.9%)	93 (68.1%)	1.29	0.86, 1.95	0.209
Heart disease					
No	1536 (26.0%)	3922 (74.0%)			
Yes	80 (43.6%)	103 (56.4%)	2.20	1.40, 3.46	0.001
High blood pressure (told by health professional)					
No	1152 (24.8%)	3126 (75.2%)			
Yes	465 (32.6%)	899 (67.4%)	1.30	0.91, 1.84	0.144
Diabetes/high blood sugar (told by health professional)					
No	1470 (26.2%)	3763 (73.8%)			
Yes	146 (32.6%)	263 (67.4%)	1.47	1.25, 1.73	<0.001
Cancer (told by health professional)					
No	1519 (26.9%)	3745 (73.1%)			
Yes	99 (24.4%)	281 (75.6%)	0.88	0.63, 1.22	0.432
Heart disease (told by health professional)					
No	1502 (26.4%)	3828 (73.6%)			
Yes	116 (31.8%)	195 (68.2%)	1.30	0.91, 1.84	0.144
Asthma (told by health professional)					
No	1372 (26.2%)	3524 (73.8%)			
Yes	246 (30.5%)	502 (69.5%)	1.24	0.99, 1.55	0.059
Chronic lung disease (told by health professional)					
No	1559 (26.3%)	3938 (73.7%)			
Yes	58 (44.6%)	88 (55.4%)	2.26	1.41, 3.64	0.001
Ulcer (told by health professional)					
No	1414 (26.3%)	3565 (73.7%)			
Yes	200 (30.1%)	459 (69.9%)	1.21	0.97, 1.51	0.097

**Table 3:** Psychological well-being over the past 30 days

Variables	Conspiracy belief group estimated mean (std. error)	Not endorsing conspiracy belief group estimated mean (std. error)	*t*	*p*
Negative well-being (higher scores better)				
Felt lonely	3.05 (0.03)	3.39 (0.03)	8.25	<0.001
Felt hopeless about the future	3.30 (0.03)	3.65 (0.01)	10.72	<0.001
Felt worthless	3.43 (0.03)	3.74 (0.01)	10.80	<0.001
A lot of psychological distress	3.15 (0.04)	3.40 (0.02)	5.83	<0.001
Feel angry and out of control	4.62 (0.04)	4.83 (0.01)	5.87	<0.001
Positive well-being (lower scores better)				
Confident	2.33 (0.02)	2.21 (0.02)	−4.33	<0.001
Optimistic	2.81 (0.04)	2.76 (0.02)	−1.21	0.235
Happy	2.44 (0.03)	2.29 (0.02)	−3.93	<0.001

Individuals who had seriously thought about committing suicide were more likely to endorse the conspiracy item (*n* = 392/1126, weighted percent = 34.1%) than individuals who had not seriously thought about committing suicide (*n* = 954/3584, weighted percent = 24.7%), odds ratio = 1.58, 95% CI =1.31, 1.91 *p* < .001, and to have greater trouble sleeping (conspiracy belief estimated mean = 2.80, std error = 0.03; not endorsing conspiracy belief group estimated mean = 2.95, std error = 0.02; higher scores indicating better sleep), *t* = 3.84, *p* < .001. Again, these two associations remained when controlling for paranoia.

### Social networks and current attachment style

It can be seen in Table 4 that a belief in conspiracy is generally associated with weaker social networks, for example, feeling less able to rely on family or friends if there is a serious problem. Current attachment styles were less secure, more avoidant, and more anxious in the individuals endorsing the conspiracy item. The significant associations were repeated controlling for paranoia, and all remained significant apart from talking on the phone/meeting friends.

**Table 4:** Social networks and current attachment style

Variables (lower scores indicate closer social networks, apart from the last two items)	Conspiracy belief group estimated mean (std. error)	Not endorsing conspiracy belief group estimated mean (std. error)	*t*	*p*
Talk on the phone or get together with relatives who do not live with you	2.76 (0.05)	2.66 (0.03)	−1.88	0.067
How much can you rely on relatives who do not live with you for help if you have a serious problem	1.83 (0.05)	1.56 (0.02)	−6.67	<0.001
Talk on the phone or get together with friends	2.67 (0.05)	2.53 (0.03)	−2.69	0.01
How much can you rely on your friends for help if you have a serious problem	2.05 (0.04)	1.79 (0.02)	−5.59	<0.001
I find it relatively easy to get close to other people. I am comfortable depending on others and having them depend on me. I do not worry about being abandoned or about someone getting too close to me	2.32 (0.04)	2.03 (0.02)	−6.33	<0.001
I am somewhat uncomfortable being close to others; I find it difficult to trust them completely and difficult to depend on them. I am nervous when anyone get too close to me	2.95 (0.04)	3.32 (0.02)	8.25	<0.001
I find that others are reluctant to get as close as I would like. I often worry that people who I care about do not love me or won’t want to stay with me. I want to merge completely with another person, and this desire sometimes scares people away	3.48 (0.03)	3.77 (0.01)	8.54	<0.001

### Childhood

Those individuals who endorsed the conspiracy belief item were more likely to have had potentially disruptive parental experiences during childhood such as not living with both biological parents, living away from home for an extended time, and often experiencing violence (see Table 5).

**Table 5:** Childhood family experiences

Variable	Conspiracy belief (*n*) (weighted percentage)	Not endorsing conspiracy belief (*n*) (weighted percentage)	Odds ratio	95% CI	*p* value
Lived with both biological parents until 16					
Yes	998 (24.6%)	2823 (75.4%)			
No	619 (31.3%)	1201 (68.7%)	1.40	1.10, 1.77	0.007
Lived away from home for at least 6 months before age 16					
No	1426 (26.1%)	3710 (73.9%)			
Yes	190 (33.6%)	313 (66.4%)	1.43	1.05, 1.96	0.024
Male head of household during childhood					
Biological father	1173 (24.7%)	3291 (75.3%)			
Adoptive/step father	200 (34.2%)	339 (65.8%)	1.58	1.25, 2.00	<0.001
Other male	85 (34.3%)	139 (65.7%)	1.59	1.00, 2.53	0.051
No male in household	156 (36.1%)	254 (63.9%)	1.72	1.29, 2.30	0.001
Female head of household during childhood					
Biological mother	1479 (26.3%)	3766 (73.7%)			
Adoptive/step mother	46 (28.9%)	109 (71.1%)	1.14	0.73, 1.78	0.549
Other female	79 (35.4%)	132 (64.6%)	1.54	1.06, 2.23	0.026
No female in household	11 (29.8%)	16 (70.2%)	1.19	0.39, 3.66	0.752
Family received government assistance for 6 months or more					
No	1333 (25.6%)	3566 (74.4%)			
Yes	234 (35.8%)	369 (64.2%)	1.63	1.33, 1.99	<0.001
When you were growing up, how often did someone in your household do any of these things to you: pushed, grabbed or shoved; threw something; slapped or hit					
Never	834 (25.4%)	2273 (74.6%)			
Rarely	296 (23.9%)	795 (76.1%)	0.93	0.75, 1.15	0.465
Sometimes	297 (31.0%)	645 (69.0%)	1.33	1.08, 1.63	0.010
Often	183 (37.2%)	295 (62.8%)	1.74	1.31, 2.32	<0.001
Woman who raised you lied a lot					
No	1503 (26.6%)	3790 (73.4%)			
Yes	78 (28.3%)	159 (71.7%)	1.09	0.69, 1.72	0.697
Man who raised you lied a lot					
No	1305 (24.9%)	3509 (75.1%)			
Yes	131 (40.9%)	220 (59.1%)	2.09	1.49, 2.92	<0.001

### Psychiatric symptoms

Every psychiatric diagnosis that we tested was significantly associated with endorsing the conspiracy belief item (see Table 6). All the analyses were repeated controlling for paranoia, and all the associations remained significant.

**Table 6:** DSM-IV disorders in past 12 months

Variable	Conspiracy belief (*n*) (weighted percentage)	Not endorsing conspiracy belief (*n*) (weighted percentage)	Odds ratio	95% CI	*p* value
Attention deficit disorder					
No	1529 (26.2%)	3926 (73.8%)			
Yes	89 (45.8%)	101 (54.2%)	2.37	1.71, 3.29	<0.001
Agoraphobia without panic disorder					
No	1548 (26.3%)	3960 (73.7%)			
Yes	70 (50.1%)	67 (49.9%)	2.92	1.82, 4.69	<0.001
Agoraphobia with panic disorder					
No	1582 (26.5%)	3991 (73.5%)			
Yes	36 (54.3%)	36 (45.7%)	3.30	1.75, 6.22	<0.001
Alcohol abuse					
No	1527 (26.3%)	3907 (73.7%)			
Yes	91 (40.6%)	120 (59.4%)	1.92	1.43, 2.56	<0.001
Alcohol dependence					
No	1571 (26.4%)	3969 (73.6%)			
Yes	47 (44.9%)	58 (55.1%)	2.27	1.49, 3.44	<0.001
Adult separation disorder					
No	1536 (26.1%)	3954 (73.9%)			
Yes	82 (55.3%)	73 (44.7%)	3.50	2.39, 5.11	<0.001
Bipolar I					
No	1579 (26.5%)	4001 (73.5%)			
Yes	39 (55.5%)	26 (44.5%)	3.46	1.94, 6.16	<0.001
Bipolar II					
No	1580 (26.5%)	3991 (73.5%)			
Yes	38 (50.4%)	36 (49.6%)	2.82	1.58, 5.04	0.001
Conduct disorder					
No	1600 (26.5%)	4012 (73.5%)			
Yes	18 (54.7%)	15 (45.3%)	3.35	1.28, 8.72	0.015
Drug abuse					
No	1560 (26.3%)	3958 (73.7%)			
Yes	58 (55.3%)	42 (44.7%)	3.46	1.922, 6.23	<0.001
Drug dependence					
No	1597 (26.5%)	4012			
Yes	21 (65.2%)	15	5.18	2.57, 10.44	<0.001
Dysthymia					
No	1504 (26.0%)	3921 (74.0%)			
Yes	114 (54.3%)	106 (45.7%)	3.38	2.56, 4.47	<0.001
Depression					
No	1310 (25.5%)	3550 (74.5%)			
Yes	308 (39.6%)	477 (60.4%)	1.91	1.51, 2.43	<0.001
Generalised anxiety disorder					
No	1450 (26.0%)	3808 (74.0%)			
Yes	168 (43.6%)	219 (56.4%)	2.20	1.83, 2.65	<0.001
Panic disorder					
No	1493 (26.1%)	3893 (73.9%)			
Yes	125 (47.0%)	134 (53.0%)	2.52	1.92, 3.30	<0.001
Social phobia					
No	1374 (25.8%)	3634 (74.2%)			
Yes	244 (38.2%)	393 (61.8%)	1.78	1.47, 2.15	<0.001
Specific phobia					
No	1316 (25.6%)	3524 (74.4%)			
Yes	302 (38.1%)	503 (61.9%)	1.79	1.51, 2.13	<0.001
PTSD					
No	1488 (26.1%)	3835 (73.9%)			
Yes	130 (42.1%)	192 (57.9%)	2.05	1.54, 2.74	<0.001
Intermittent explosive disorder					
No	1440 (25.9%)	3825 (74.1%)			
Yes	178 (44.2%)	202 (55.8%)	2.27	1.73, 2.98	<0.001

## Discussion

The causes of events are typically opaque. Organised conspiracies do occur and are sometimes uncovered, often after protracted denial by the perpetrators. A certain level of scepticism towards official explanations of events may therefore be warranted, just as sometimes it may be adaptive to mistrust the intentions of others. The results from this national survey, however, indicate that a general tendency to see conspiracies underlying events is associated with a wide range of negative life circumstances. Levels of unhappiness, negative emotions, and isolation are greater in those who view the world in terms of conspiracies.

Many of the factors associated with the belief in conspiracies are similar to those previously observed in association with paranoia in both psychiatric and non-psychiatric populations; for example, paranoia has been associated with social conditions characterised by victimisation and powerlessness [19] and with low self-esteem and negative emotion [6]. Current attachment style has also been linked to paranoia in the NCS-R [20]. However, although we found an association between conspiracy belief and paranoia, the indicators of distress associated with a conspiracy world view at the individual level were maintained even when paranoia was controlled for in our analyses. Further, conspiracy beliefs were not associated with a particular age, whereas paranoid ideation is greater in youth. We reported all the tests we made of conspiracy beliefs with variables from the NCS-R dataset, so it is remarkable how the pattern of significant findings is so wide-ranging and consistent, indicating the potential importance of conspiracy theories for understanding both mental health and social cohesion in modern societies.

There are clear limitations to the study. First, we note that the cross-sectional design prevents inferences concerning causality. In this report we were simply establishing correlates. Hence, it cannot be determined whether the conspiracy views have formed as a way of managing difficult life circumstances or have led to such problems or whether we are simply seeing many related markers of a marginalised group. It also could be that we are simply observing a phenomenon better explained by an unmeasured confounder. Other approaches, part of a process of triangulation, are needed to understand the nature of these associations, for example, longitudinal, experimental, and interventionist methods [21–23]. Second, the assessment of conspiracy beliefs relied on one item only, albeit one with clear face validity, though we think there are multiple compensations provided by its use in a large population that was assessed on a wide variety of social, psychological, and psychiatric variables. Individuals who endorse one conspiracy theory are highly likely to believe in others (even contradictory ones), and psychometric research confirms a general tendency towards conspiracy ideation [24, 25], which may be captured by this item. Nonetheless future work would clearly benefit from a detailed assessment of conspiracy thinking. Third, although the study concerns a general tendency to see conspiracies behind events which has been observed in previous research, it could well be that isolated single conspiracy beliefs may serve different functions or that there are fluctuations in time in such a worldview. Finally, the survey was conducted over 10 years ago in one country and it is obviously not implausible to think that the prevalence and nature of such a worldview may have changed in the intervening years. We recommend repeated monitoring of levels of mistrust in the general population.

**Fig. 1:**
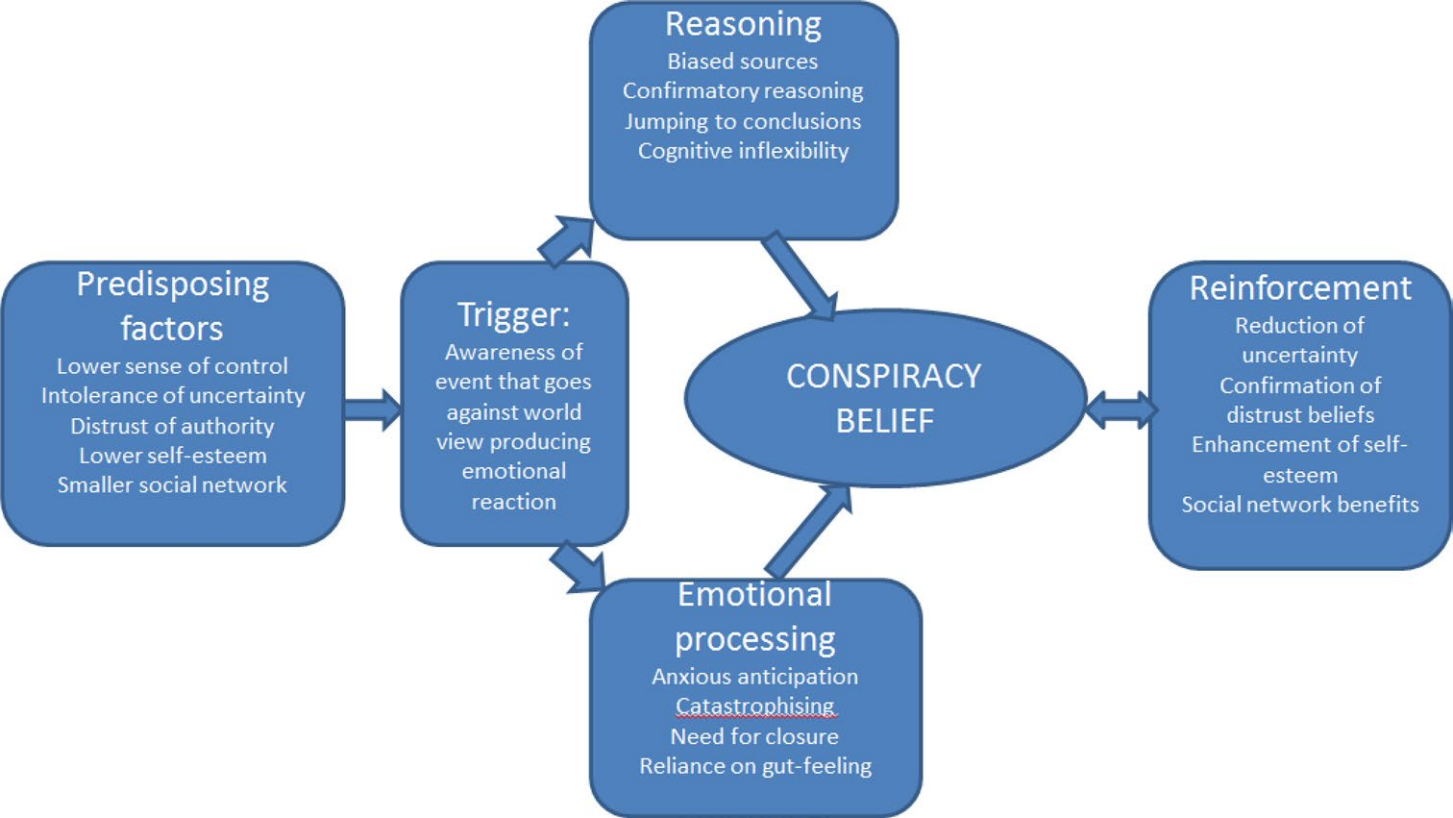
A psychological conceptualisation of conspiracy beliefs

Conspiracy world views clearly develop from a complex interaction of factors. Our view at the psychological level of explanation (summarised in Fig. 1) is that low self-esteem, distrust of authority, and smaller social networks, often in the context of social marginalisation, develop in the early years so that, in young adulthood, they provide the context for understanding the occurrence of specific world events that are threatening or inconsistent with expectations. Both affective and reasoning processes contribute to the occurrence of a specific conspiracy belief that then has multiple short-term benefits, including a reduction in uncertainty and also access to social networks of like-minded people (especially in the age of the internet). The current survey results certainly support the presence of low self-esteem, anxiety, and marginalisation in those holding a conspiracist view of world events.

Conspiracy theories are sufficiently definable, measureable, and observable to be suitable for scientific investigation. By developing methods of investigation, by advancing the understanding of their causes, and by studying their impact on the individual, social networks, and society as a whole, it may be possible to gain not only a substantial, robust, and unique understanding of these kinds of beliefs but also provide a framework for conceptualising the individual and social significance of belief systems in general.
